# Endangered mammal occurrence records from Environmental Impact Assessment and post-EIA monitoring in the Republic of Korea

**DOI:** 10.3897/BDJ.14.e212750

**Published:** 2026-10-13

**Authors:** Jungkwon Kim, Hae-Ran Kim, Minho Chang, Nanghee Kim

**Affiliations:** 1 National Institute of Ecology, Seocheon, Republic of Korea National Institute of Ecology Seocheon Republic of Korea

**Keywords:** biodiversity database, ecological survey, environmental assessment, Mammalia, post-EIA monitoring, species occurrence

## Abstract

**Background:**

Environmental Impact Assessment (EIA) and post-EIA monitoring generate substantial biodiversity information through ecological surveys conducted before, during and after development projects. In the Republic of Korea, these records have accumulated in individual assessment reports, but their reuse has been limited because occurrence information is dispersed across documents and is not generally available in a structured format. Compiling these records into a standardised dataset can improve access to historical biodiversity information and support research, conservation planning and environmental assessment.

**New information:**

The dataset comprises 5,000 occurrence records of six endangered mammal taxa documented in EIA and post-EIA monitoring reports in the Republic of Korea. The records cover surveys conducted from March 2007 to June 2023 and include taxonomic information, survey dates, detection methods and generalised geographic coordinates. Occurrence locations presented on maps in the original reports were manually digitised and the records were standardised using Darwin Core terms. The dataset integrates occurrence records from the assessment, construction and operation phases and is openly available through the Global Biodiversity Information Facility (GBIF).

## Introduction

Environmental Impact Assessment (EIA) is a decision-support process used to identify and evaluate the potential environmental consequences of proposed development before implementation (Morgan 2012). Ecological surveys, conducted as part of EIA, generate species occurrence information that supports the assessment of potential biodiversity impacts and the planning of avoidance, minimisation, restoration and offset measures (King et al. 2012, GBIF Secretariat and IAIA 2020). As such surveys are repeatedly conducted for development projects across different regions and habitat types, EIA reports collectively contain a substantial volume of biodiversity data with potential value beyond the individual assessments for which the data were originally collected (King et al. 2012, GBIF Secretariat and IAIA 2020).

In addition to assessment-stage surveys, post-EIA monitoring during construction and operation extends the temporal coverage of species occurrence information by providing repeated records beyond the assessment phase. Previous studies in the Republic of Korea have demonstrated the value of biodiversity records contained in EIA and post-EIA monitoring reports (Lee et al. 2024, Seo et al. 2024, Kim et al. 2025).

Despite this potential, biodiversity records generated through EIA frequently remain embedded within individual reports and are not readily available as structured, reusable data. Limited accessibility, inconsistent data structures and gaps in data availability can hinder the effective use of biodiversity information in spatial planning and impact assessment (King et al. 2012, Underwood et al. 2018, Telletxea et al. 2025, Telletxea et al. 2026). International guidance recommends that biodiversity records generated through environmental assessment be standardised and made accessible (King et al. 2012, GBIF Secretariat and IAIA 2020). Recent studies have also demonstrated the mobilisation of such records from environmental assessment documents (Telletxea et al. 2025, Telletxea et al. 2026). When such records are published as open datasets, they can be more readily discovered, reused and integrated with other biodiversity data, consistent with the FAIR principles of findability, accessibility, interoperability and reusability (Wilkinson et al. 2016).

Occurrence information on endangered species is particularly important because it can be used to assess potential impacts of development projects and to plan avoidance and mitigation measures (GBIF Secretariat and IAIA 2020, Seo et al. 2024). In particular, some mammal species with large home ranges and high mobility may be affected by habitat loss and fragmentation and by impeded movement associated with infrastructure development, making their spatial occurrence information useful for assessing development impacts and evaluating mitigation measures (Benítez-López et al. 2010, Rytwinski and Fahrig 2012). In addition, mammal occurrence information can accumulate across different project phases, including the assessment, construction and operation stages, through EIA and post-EIA monitoring. Previous research on road-construction projects in the Republic of Korea also showed that mammals accounted for a substantial proportion of endangered wildlife occurrence records (Kim et al. 2025).

Accordingly, this study aimed to compile and standardise occurrence records of endangered mammals documented in publicly available EIA and post-EIA monitoring reports from selected development projects in the Republic of Korea and to make these records available as a reusable biodiversity dataset. The dataset integrates occurrence information recorded during the assessment, construction and operation phases of development projects. This data paper describes the data sources, compilation and quality-control procedures and provides the resulting dataset for reuse in biodiversity research, conservation and environmental assessment.

## General description

### Purpose

The dataset was developed to improve the accessibility and reuse of endangered mammal occurrence records generated through Environmental Impact Assessment and post-EIA monitoring in the Republic of Korea. By compiling records dispersed across individual reports into a structured dataset, this work provides a resource for biodiversity research, species distribution studies, conservation planning and environmental assessment.

### Additional information

The dataset represents occurrence records documented in Environmental Impact Assessment and post-EIA monitoring reports and should not be interpreted as the results of a systematic or comprehensive survey of endangered mammal distributions in the Republic of Korea. No occurrence records from other published sources or external biodiversity databases were included. Its spatial and temporal coverage reflects the locations and survey periods of the development projects included in the source reports.

## Project description

### Title

Compilation of endangered mammal occurrence records from Environmental Impact Assessment and post-EIA monitoring reports in the Republic of Korea

### Personnel

The project was conducted by researchers at the National Institute of Ecology (NIE), Republic of Korea. The authors participated in data extraction, data curation, validation, dataset design and publication.

### Study area description

The occurrence records were extracted from EIA and post-EIA monitoring reports associated with development projects across the Republic of Korea, covering diverse regions and habitat types, including forests, wetlands, rivers, agricultural areas, coastal areas and urban environments.

### Design description

Development projects for which the EIA consultation process was completed between 2010 and 2019 and for which post-EIA monitoring had been completed by 2024 were considered for inclusion. Amongst these, projects in which endangered mammals were documented in either the EIA or post-EIA monitoring reports were included in the final dataset. As ecological surveys conducted during the assessment phase may precede completion of the EIA consultation process, occurrence records from these projects may include survey dates earlier than 2010. Road-construction projects were excluded because occurrence records from these projects had been compiled and published separately in a previous study (Kim et al. 2025). EIA and post-EIA monitoring reports for the selected projects were reviewed to compile occurrence records of endangered mammals.

### Funding

This work was supported by a grant from the National Institute of Ecology (NIE), funded by the Ministry of Climate, Energy and Environment of the Republic of Korea (NIE-C-2026-19) and by the Korea Environment Industry and Technology Institute (KEITI) (NIE-C-2024-61).

## Sampling methods

### Study extent

Publicly available Environmental Impact Assessment (EIA) reports and reports on follow-up surveys of environmental impacts (hereafter, post-EIA monitoring reports) for the selected projects were obtained through the national EIA information system (Environmental Impact Assessment Support System (EIASS) 2026).

### Sampling description

Occurrence records of endangered mammals were extracted from ecological survey results documented in EIA and post-EIA monitoring reports prepared in accordance with the Environmental Impact Assessment Act (Republic of Korea 2025). Records from the assessment, construction and operation phases were included where available. Each occurrence record represents a detection of a taxon documented for a specific development project, project phase and survey event. Records from different development projects, project phases or survey events were retained as separate occurrences, even when the same taxon was recorded at the same location. Detections were based on direct observations, indirect evidence such as tracks, scat, feeding signs and habitat evidence, as well as camera-trap records and other survey methods reported in the original documents. Species names, survey dates, detection methods and occurrence locations were extracted from the reports. Occurrence locations shown on maps in the source reports were manually digitised using Google Earth Pro (version 7.3.6.9345). The initially digitised coordinates were derived from mapped locations rather than transcribed from original field GPS coordinates and their spatial precision, therefore, reflects the scale and level of detail of the source maps. To protect sensitive location information for legally protected endangered mammals while retaining the spatial utility of the dataset, the published coordinates were subsequently generalised to the centre of the corresponding 1-km grid cell. Original, non-generalised coordinates are not included in the publicly available dataset.

### Quality control

Species names and taxonomic information were reviewed and standardised according to the National Species List of Korea (National Institute of Biological Resources 2026). Geographic coordinates were cross-checked against occurrence locations shown in the source maps and spatial inconsistencies and data-entry errors were identified and corrected. Survey dates and detection methods were standardised according to the information available in the source reports. Duplicate records were defined as repeated records of the same taxon at the same location within the same development project, project phase and survey event. These records were consolidated and retained as a single occurrence record. Metadata and field names were standardised using Darwin Core terms (Wieczorek et al. 2012) prior to publication through the Global Biodiversity Information Facility (GBIF).

### Step description

The dataset was developed through five sequential steps: (1) selection of development projects and retrieval of the corresponding EIA and post-EIA monitoring reports; (2) extraction of endangered mammal occurrence records and associated metadata; (3) manual digitisation of occurrence locations shown on maps using Google Earth Pro; (4) standardisation and quality control of taxonomic, spatial, temporal and observational information, including coordinate validation, spatial generalisation using QGIS Desktop and standardisation using Darwin Core terms; and (5) publication of the standardised dataset through the Global Biodiversity Information Facility (GBIF).

## Geographic coverage

### Description

The dataset includes endangered mammal occurrence records documented in EIA and post-EIA monitoring reports for development projects across the Republic of Korea. The geographic distribution of the occurrence records is shown in Fig. 1.

### Coordinates

34.618683 and 38.066860 Latitude; 126.380588 and 129.372188 Longitude.

## Taxonomic coverage

### Description

The dataset comprises occurrence records for six mammal taxa designated as Endangered Wildlife under the Enforcement Rule of the Wildlife Protection and Management Act of the Republic of Korea (Republic of Korea 2026). The number and proportion of records for each taxon are summarised in Table 1.

### Taxa included

**Table:** 

Rank	Scientific Name	
species	*Lutra lutra*	
species	*Prionailurus bengalensis*	
subspecies	*Pteromys volans aluco*	
species	*Martes flavigula*	
species	*Mustela nivalis*	
species	*Plecotus ognevi*	

## Temporal coverage

**Data range:** 2007-3-01 – 2023-6-01.

### Notes

The temporal coverage is based on the survey dates recorded in the EIA and post-EIA monitoring reports. These survey dates are distinct from the year in which the EIA consultation process was completed, which was used as a criterion for project selection. Values in the eventDate field were standardised to year or year-month according to the temporal resolution available in the source reports.

## Usage licence

### Usage licence

Creative Commons Public Domain Waiver (CC-Zero)

## Data resources

### Data package title

Endangered mammal occurrence records from Environmental Impact Assessment and post-EIA monitoring in the Republic of Korea

### Resource link

https://doi.org/10.15468/2w6j8g

### Number of data sets

1

### Data set 1.

#### Data set name

Endangered mammal occurrence records from Environmental Impact Assessment and post-EIA monitoring in the Republic of Korea

#### Data format

Darwin Core Archive (DwC-A)

#### Character set

UTF-8

#### Download URL

https://naris.science.go.kr/kbif/archive.do?r=eia_mammals_korea

#### Description

The dataset contains 5,000 occurrence records representing six endangered mammal taxa documented in EIA and post-EIA monitoring reports from 73 development projects in the Republic of Korea (Kim 2026). The records include taxonomic, spatial, temporal and observational information. To protect sensitive location information for legally protected endangered mammals, the published coordinates were generalised to the centre of the corresponding 1-km grid cell. The dataset is published through the Global Biodiversity Information Facility (GBIF).

**Data set 1.** 

Column label	Column description
occurrenceID	A unique identifier assigned to each occurrence record, composed of the institution code, project-type code, project identifier, project phase and sequential occurrence number. Project phases are coded as EIA (assessment), CON (construction) and OPR (operation).
institutionCode	The code of the institution responsible for publishing and managing the dataset; all records are assigned to the National Institute of Ecology (NIE).
basisOfRecord	The nature of the occurrence record, classified as HumanObservation or MachineObservation according to the detection method.
occurrenceStatus	A statement indicating whether the taxon was present or absent at the recorded location; all records in this dataset are recorded as present.
eventDate	The year or year-month in which the ecological survey was conducted, according to the temporal information available in the source report.
scientificName	The standardised scientific name of the recorded taxon.
vernacularName	The English vernacular name of the recorded taxon.
kingdom	The taxonomic kingdom of the recorded taxon.
phylum	The taxonomic phylum of the recorded taxon.
class	The taxonomic class of the recorded taxon.
order	The taxonomic order of the recorded taxon.
family	The taxonomic family of the recorded taxon.
genus	The genus of the recorded taxon.
specificEpithet	The specific epithet of the recorded taxon.
infraspecificEpithet	The infraspecific epithet of the recorded taxon, where applicable.
taxonRank	The taxonomic rank of the recorded taxon, recorded as species or subspecies.
samplingProtocol	The standardised survey or detection method associated with each occurrence record, including direct observation, indirect signs such as tracks and scat, camera-trap survey and other documented detection methods.
decimalLatitude	The latitude of the centre of the 1-km grid cell representing the generalised occurrence location, in decimal degrees using the WGS84 geodetic datum.
decimalLongitude	The longitude of the centre of the 1-km grid cell representing the generalised occurrence location, in decimal degrees using the WGS84 geodetic datum.
geodeticDatum	The geodetic datum used for the geographic coordinates; all records use WGS84.
country	The country in which the occurrence was recorded; all records are from the Republic of Korea.
countryCode	The ISO 3166-1 alpha-2 country code for the occurrence location; KR indicates the Republic of Korea.
dynamicProperties	Additional information on the legal conservation status of the recorded taxon in the Republic of Korea, expressed as endangeredWildlifeClass (Class I or Class II).
coordinateUncertaintyInMetres	The coordinate uncertainty associated with the generalised occurrence location, expressed in metres; all records were assigned a value of 800 m to conservatively account for displacement within the 1-km grid cell and positional uncertainty from manual digitisation.
dataGeneralisations	A description of spatial generalisation applied to the occurrence coordinates; coordinates were generalised to the centre of the corresponding 1-km grid cell to protect sensitive endangered-species location information.
informationWithheld	A description of information withheld from the published dataset; original, non-generalised coordinates are not included.

## Figures and Tables

**Figure 1.**
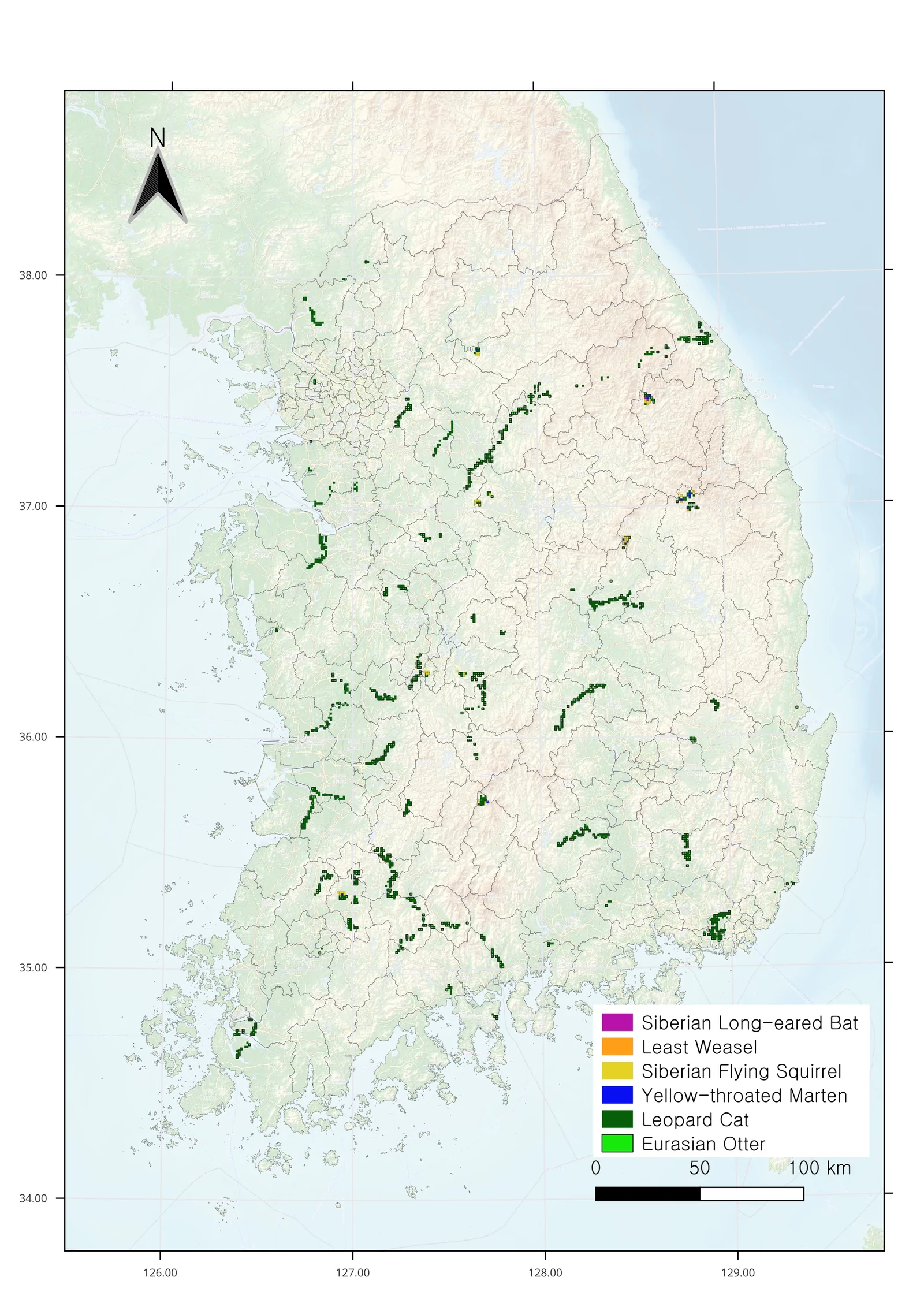
Geographic distribution of endangered mammal occurrence records documented in EIA and post-EIA monitoring reports in the Republic of Korea. Generalised occurrence locations are displayed as 1-km grid cells.

**Table 1.** Number and proportion of occurrence records for the six endangered mammal taxa included in the dataset.

Scientific name	Common name	Endangered wildlife class	Number of records	Percentage (%)
*Lutra lutra*	Eurasian Otter	I	2,750	55.00
*Prionailurus bengalensis*	Leopard Cat	II	1,984	39.68
*Pteromys volans aluco*	Siberian Flying Squirrel	II	196	3.92
*Martes flavigula*	Yellow-throated Marten	II	65	1.30
*Mustela nivalis*	Least Weasel	I	3	0.06
*Plecotus ognevi*	Siberian Long-eared Bat	II	2	0.04
Total			5,000	100.00
